# The Association between Bisphenol A Exposure and Obesity in Children—A Systematic Review with Meta-Analysis

**DOI:** 10.3390/ijerph16142521

**Published:** 2019-07-15

**Authors:** Ka Young Kim, Eunil Lee, Yanghee Kim

**Affiliations:** 1 Department of Nursing, College of Nursing, Gachon University, 191 Hambakmoeiro, Yeonsu-gu, Incheon City 21936, Korea; 2 Department of Preventive Medicine, College of Medicine, Korea University, 73 Inchon-ro, Seongbuk-gu, Seoul 02841, Korea

**Keywords:** Bisphenol A (BPA), obesity, childhood, meta-analysis, systematic review

## Abstract

Bisphenol A (BPA) is an environmental chemical that has adverse effects on health, probably causing childhood obesity. However, this association remains controversial, and it is difficult to find evidence for direct causality between environmental exposure and disease using epidemiological studies. In this study, we sought to elucidate the possible causality between BPA exposure and childhood obesity by conducting two meta-analyses showing bidirectional associations, including exposure effect by obesity and obesity risk by exposure. Articles published up to September 2017 were searched in PubMed, Embase, and Cochrane Library. We evaluated observational studies that included measurements of urinary BPA concentration and BMI or body weight. Of 436 articles, a total of 13 studies were included in the meta-analysis. Two meta-analyses were performed to investigate the association between BPA exposure and childhood obesity. The results showed that the relatively high-exposed group had a significantly higher risk of childhood obesity than the relatively low-exposed group (odds ratio = 1.566, 95% confidence interval [CI]: 1.097 to 2.234, *p* = 0.014). However, the obese group showed no significant difference in the BPA concentration when compared to the normal group (standardized mean difference = 0.166, 95% CI: −0.121 to 0.453, *p* = 0.257). This study suggested possible causality between BPA exposure and childhood obesity using data from epidemiological studies and showed that BPA exposure itself increased the risk of obesity in children.

## 1. Introduction

Endocrine disrupting chemicals (EDCs) are substances that interfere with the endocrine system and metabolism, influencing public health as well as the health of individuals [1]. In particular, bisphenol A [BPA; 2,2-bis(4-hydroxyphenyl)propane] is widely used in food cans, reusable bottles such as baby bottles, food storage containers, and composites and sealants in dentistry [2,3]. BPA exposure caused by persistent usage of such objects in daily life is known to result in various adverse health effects. Furthermore, early exposure of BPA in the prenatal period and during childhood is associated with developmental and metabolic disorders [4,5].

According to the world health organization, obesity is among the most important global public health issues [6]. Obesity is associated with genetic, environmental, psychological, social, and economic factors; however, the causes and mechanisms are not fully understood yet [7,8]. Furthermore, the prevalence of obesity is increasing rapidly in children aged 2–18 years [9]. Obese children are also likely to be obese in adulthood, and are more likely to develop metabolic disorders, cardiovascular diseases, and cancer at an early age [10,11].

In particular, early life exposure to BPA is known to be associated with childhood obesity [12,13]. Despite the close association between obesity and environmental factors, the actual impact of this connection tends to be underestimated, owing to the difficulties in proving a causal association. Moreover, although it is essential to elucidate the associations between BPA exposure and obesity, the results have been controversial. Many studies have demonstrated that BPA exposure is associated with childhood obesity [1,14], while some studies reported no association between BPA exposure and childhood obesity [15,16]. No epidemiological studies have shown a direct causality between BPA exposure and childhood obesity. Therefore, in this study, we sought to elucidate the relatively possible causality between BPA exposure and childhood obesity by conducting systematic reviews with two meta-analyses, showing bidirectional associations, including exposure effect by obesity and obesity risk by exposure.

## 2. Materials and Methods 

### 2.1. Literature Search 

This study was conducted in accordance with the PRISMA guideline [17]. We searched PubMed, Embase, and Cochrane Library databases to identify all relevant studies published in English on the association between BPA exposure and childhood obesity before September 30, 2017. The terms for the search were used as follows: (bisphenol) AND (obese* OR weigh* OR fat* OR overweight* OR body mass index OR BMI OR waist circumference) AND (child* OR pediatric OR pueril* OR juvenile or infant* or newborn or neonatal or baby or toddler or suckling or adolescent*).

### 2.2. Selection Criteria 

For meta-analysis, we selected human studies with a wide age span ranging from birth to adolescence with a cross-sectional, case control, or cohort design that included measurement of urinary BPA concentration and BMI or body weight, and reported on associations such as relative risk (RR) or odds ratio (OR) for obesity between relatively high-exposed and relatively low-exposed groups, or reported the mean difference in urinary BPA levels between the obese and the normal group. To distinguish the relatively high-exposure and the relatively low-exposure groups and obese and normal groups, we performed the meta-analysis according to the concept used in each paper. Articles in the form of a commentary, editorial, review, or meta-analysis were excluded from this study.

### 2.3. Data Extraction

Two authors (K.Y. Kim and Y. Kim) screened relevant data independently, including the first author, year of publication, study type, participants, age of participants, sample size per each group, location, urinary BPA concentration, measurement units, adjusted variables, and outcomes. Furthermore, all authors discussed to resolve any disagreements. To demonstrate the association between BPA and obesity in childhood, we extracted all measurement data for urinary BPA exposure levels, BMI, and body weight. Then, BMI or body weight data were extracted for the relatively high-exposed and the relatively low-exposed groups, and BPA exposure was extracted for the obese and normal groups.

### 2.4. Quality Evaluation

Methodological quality control was conducted on the selected final 13 papers that included case control, cross-sectional, and cohort studies. Quality evaluation was performed using the Newcastle–Ottawa quality assessment scale (NOS), a tool used for assessing the quality of non-randomized studies [18]. 

### 2.5. Data Analysis 

The obesity risk estimates between the relatively high-exposure group and the relatively low-exposure group and the difference in BPA levels between the obese group and the normal group were analyzed by meta-analysis using the comprehensive meta-analysis (CMA) software version 3 (Biostat Inc., Englewood, NJ, USA). We assessed the heterogeneity using the *I^2^* method and evaluated publication bias using funnel plots. *P* < 0.05 indicated a significant difference.

## 3. Results

### 3.1. Characteristics of the Studies 

**Figure 1:**
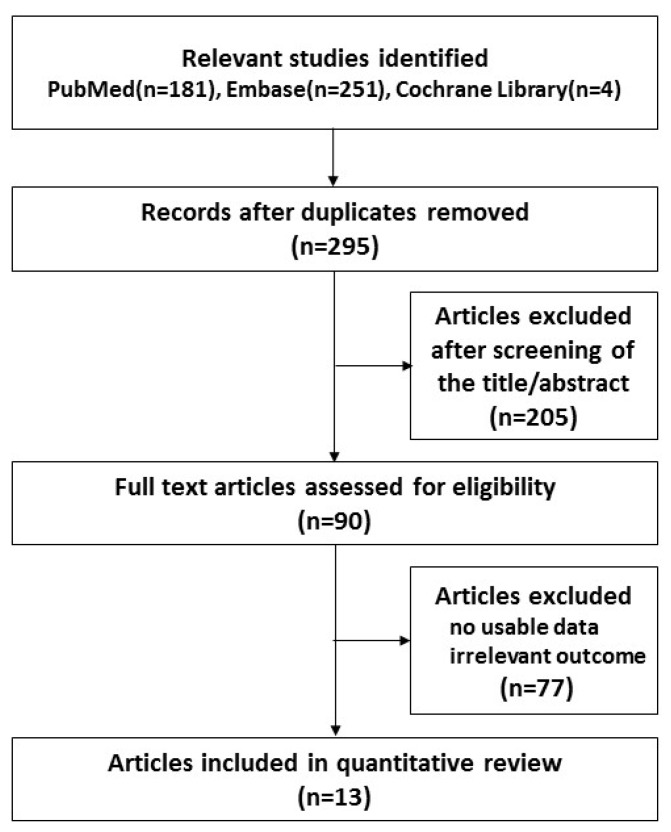
Flow chart of study selection in the meta-analysis.

For the literature search, we initially identified 436 articles (Figure 1). After discarding 141 duplicate papers using the Endnote reference database, 295 articles were selected. Next, 205 articles were excluded based on the titles and abstracts, primarily because they were molecular studies, adult studies, and animal studies. Then, ninety articles were eligible for full-text review. After close examination, 77 publications were excluded because they lacked BPA exposure data or outcome information. Finally, a total of 13 articles were identified and included in the meta-analysis. Table 1 shows the characteristics of the 13 included studies. There were eight cross-sectional studies, three cohort studies, and two case-control studies; three of the studies were pilot studies. All the studies included in the meta-analysis were published between 2012 and 2017. The number of participants ranged from 54 to 2838. The participants were aged between 14 months and 19 years. The outcomes and the quality assessment of the 13 studies are shown in Table 1.

### 3.2. Meta-Analysis of Childhood Obesity by Exposure Groups 

**Figure 2:**
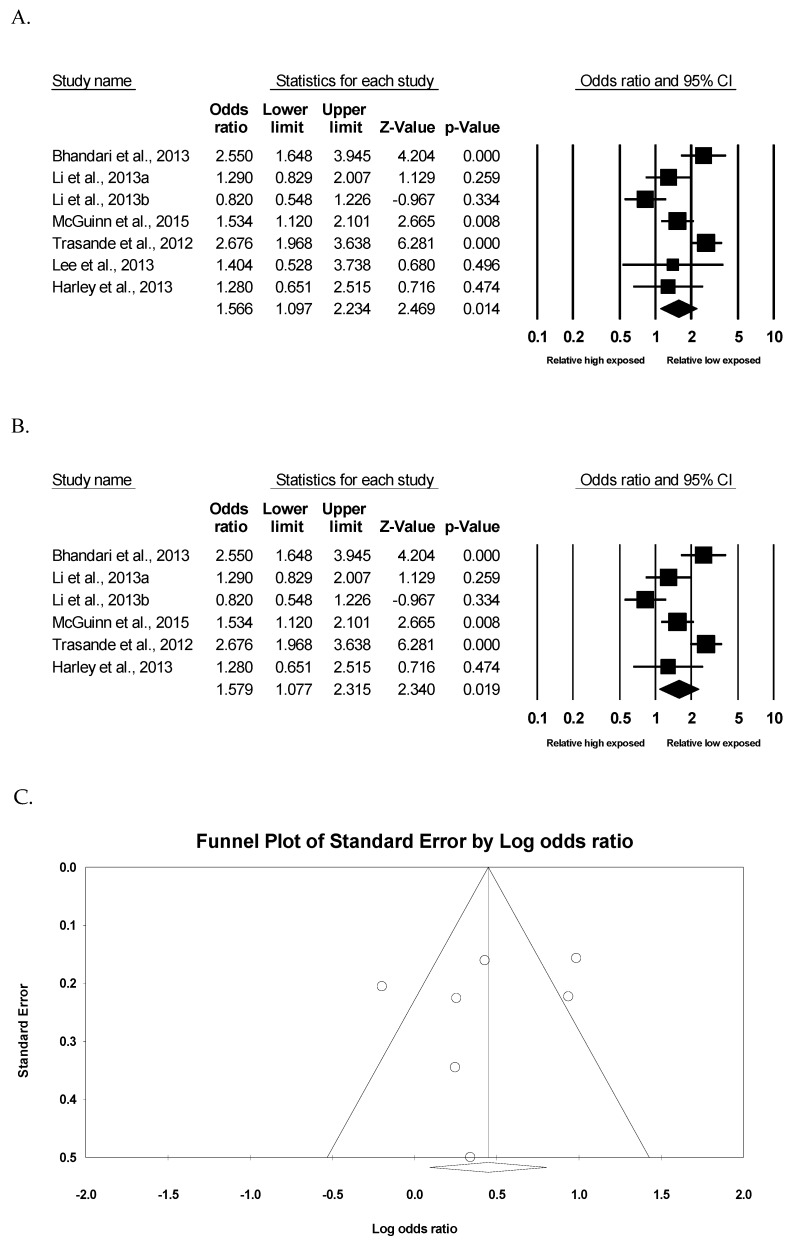
Meta-analysis of childhood obesity by exposed group. (**A**) Forest plot of seven data on the risk of childhood obesity by exposed group. (**B**) Forest plot of six data, except one pilot study on the risk of childhood obesity by exposed group. (**C**) Funnel study in the meta-analysis on the association between BPA exposure and childhood obesity.

To investigate the association between BPA exposure and childhood obesity, two meta-analyses were performed in this study. To begin with, in six of the 13 studies, the health risks of being obese were analyzed according to the relatively low-exposed group (reference group) and the relatively high-exposed group in each study. As there was significant heterogeneity (*I*^2^ = 77.8%, *p* <0.001) among studies, the random effect model was used to compute the effect size. As observed in Figure 2A, meta-analysis showed that the relatively high-exposed group had a significantly higher risk of childhood obesity than the relatively low-exposed group (OR = 1.566, 95% confidence interval [CI]: 1.097 to 2.234, *p* = 0.014). Furthermore, the relatively high-exposure group still had a significantly higher risk of childhood obesity in five studies, excluding one pilot study due to the small sample size (OR = 1.579, 95% CI: 1.077 to 2.315, *p* = 0.019) (Figure 2B). As observed in Figure 2C, the funnel plot did not show an obvious risk of publication bias (Egger’s test *p* = 0.528).

### 3.3. Meta-Analysis of BPA Exposure by Childhood Obesity 

**Figure 3:**
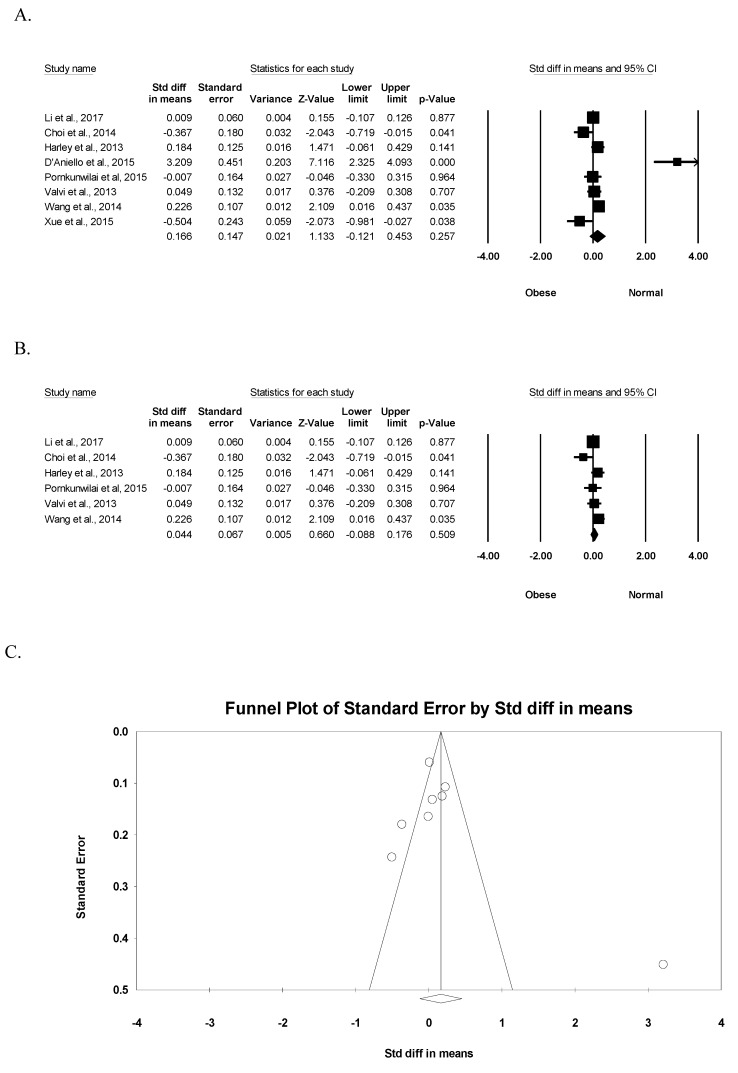
Meta-analysis of BPA exposure by childhood obesity. (**A**) Forest plot of eight studies on the exposed BPA concentration by childhood obesity. (**B**) Forest plot of six studies, except two pilot studies, on the exposed BPA concentration by childhood obesity. (**C**) Funnel study in the meta-analysis on the association between BPA exposure and childhood obesity.

To demonstrate the association between BPA exposure and childhood obesity, the difference in BPA exposure was analyzed according to obese and normal groups. Significant heterogeneity (*I*^2^ = 89.1%, *p* <0.001) was observed among studies, and the random-effect model was used to compute the pooled effect size. Meta-analysis showed that the obese group showed no significant difference in the exposed BPA concentration when compared to the normal group (standardized mean difference (SMD) = 0.166, 95% CI: −0.121 to 0.453, *p* = 0.257) in the eight relevant studies (Figure 3A). In six studies, except two pilot studies, the results also indicated that the obese group had no significant difference in the exposed BPA level (SMD = 0.044, 95% CI: −0.088 to 0.176, *p* = 0.509) (Figure 3B). As observed in Figure 3C, the funnel plot did not show an obvious risk of publication bias (Egger’s test *p* = 0.408).

## 4. Discussion

This study has elucidated the possible causality between BPA exposure and childhood obesity. The results demonstrated a significant association of childhood obesity by BPA exposure, while no significant association of BPA exposure by childhood obesity was observed. Several studies have demonstrated the association between BPA exposure and childhood obesity [1,14,19]. However, there is no consensus owing to the difficulties and limitations of epidemiological evidence [15,16]. In particular, it is difficult to prove a causal relationship between environmental exposure and disease. Therefore, to assess the impact of BPA exposure on the risk of childhood obesity, two-side meta-analyses were performed in this study. It is well known that BPA is commonly used in reusable baby bottles, food storage containers, and beverage cans [3]. So, children can be easily exposed in everyday life, and children who eat a lot of food can more easily be exposed to BPA, therefore, this tends to lead to obesity [3]. However, this study showed that BPA exposure itself would lead to childhood obesity. The results showed that the relatively high BPA exposure group had a significantly higher risk of childhood obesity than the relatively low BPA exposure group, as shown in Figure 2. However, the obese group did not show significant difference in BPA concentration compared to the normal group, as shown in Figure 3. Therefore, this study suggested that BPA exposure itself leads to childhood obesity via two meta-analyses, showing bidirectional associations. Several studies have shown that BPA increased the risk of metabolic disorders and obesity by changing the endocrine-metabolic pathways in adipose tissue [5,20]. BPA increases the number and size of adipocytes by regulating the expression of genes such as *FABP4*, *CD36*, and *PCSK1* [5]. Some studies showed that BPA exposure could decrease the release of adiponectin and adipokine with protective features against obesity-related metabolic syndrome [8,21]. Prolonged exposure to low doses of BPA impairs adipogenesis and causes adipocyte metabolic dysfunction, increasing the risk of developing obesity-related disease [22]. BPA exposure is inevitable in daily life, and the effects of BPA exposure have been analyzed in patients of all ages including neonates, children, and adults [23]. People are exposed to BPA via dietary ingestion, inhalation, and dermal contact in daily life [12]. However, prolonged or high exposure to BPA during the early life may result in more permanent adverse effects, and increase the risk of chronic diseases, such as metabolic and cardiovascular disease, in adult life [8,24]. Therefore, it is important to elucidate the possible causal association between BPA exposure and childhood obesity by conducting two systematic reviews with meta-analyses.

However, this study has some limitations because the systematic review with meta-analysis was performed with limited research papers and there are difficulties of epidemiological evidence. First, although age and sex are important factors in the developmental stages, this study used the broad age criteria of birth to preschool as the childhood period without considering the sex for meta-analysis [16,25,26]. Current research to evaluate BPA exposure and childhood obesity according developmental stage is very limited, and sometimes shows inconsistent results [19,27]. Furthermore, BPA is still one of many factors associated with obesity, and other predisposing factors should be considered to demonstrate the cause of obesity. Therefore, we tried to elucidate the tendency for overall early exposure via the mean difference within each paper. However, further study is needed according to each developmental stage and sex. Second, the criteria for obesity were not consistent across the included studies. In some studies, the obese group was classified as being over the 85^th^ percentile of the BMI, while in some, they were classified as being over the 90^th^ percentile, and in others, as being over the 95^th^ percentile. Therefore, in this study, the obese group was included as long as it was classified as the obese group within each study. However, further research is needed by including studies with identical percentile criteria.

## 5. Conclusions

This study elucidated the possible causal association between BPA exposure and childhood obesity by conducting two systematic reviews with meta-analyses. The epidemiological data suggested that BPA exposure itself increased the risk of obesity in children. Further investigation is needed for causal association between BPA exposure and obesity on each developmental stage and sex, and elucidate the related mechanisms, to have better knowledge for the prevention and management of environmental disease.

## Figures and Tables

**Table 1:** Characteristics of the 13 included studies on the association between BPA exposure and childhood obesity.

Study	Study Type	Participants	Location	Age	Outcome	Quality *
Bhandari et al., 2013 [26]	Cross-sectional	2200	USA	6–18 y	Positive association was observed between BPA levels and obesity, independent of age, sex, race/ethnicity, education, physical activity, serum cotinine, and urinary creatinine.	9
Lee et al., 2013 [28]	Cohort (pilot study)	80	Korea	7–8 y	BPA exposure affected hormone level such as estradiol, androstenedione, testosterone, insulin, and homeostasis model assessment of insulin resistance index.	7
Li et al., 2013 [25]	Cross-sectional	1326	China	9–12 y	A higher urine BPA level (≥2 µg/L) increased 2-fold more in the risk of obesity.	9
McGuinn et al., 2015 [29]	Cross-sectional	987	USA	12–19 y	BPA was associated with early onset of menarche and the association was modulated by obesity status.	9
Trasande et al., 2012 [30]	Cross-sectional	2838	USA	6–19 y	Urinary BPA concentration was significantly associated with obesity in children and adolescents.	9
Harley et al., 2013 [27]	Cohort	311	USA	5–9 y	Urinary BPA concentration at 5 years was not associated with BMI, but BPA concentration at 9 years was positively associated with BMI.	8
D’Aniello et al., 2015 [14]	Case-control (pilot study)	54	Italy	5–16 y	Free and total BPA levels were associated with the increase in BMI and conjugated BPA was related to the decrease in BMI.	6
Li et al., 2017 [16]	Cross-sectional	1860	USA	8–19 y	Higher BPA levels were related to elevated lean body mass in boys, but not in girls, while higher BPA was associated with increased fat mass in girls, but not in boys.	9
Xue et al., 2015 [31]	Case-control (pilot study)	76	India	2–14 y	Target chemicals including BPA had no significant association with childhood obesity.	7
Choi et al., 2014 [32]	Cross-sectional	127	Korea	6–14 y	BPA had no significant association with childhood obesity.	8
Wang et al., 2014 [15]	Cross-sectional	666	China	9–12 y	BPA level was not associated with age, and there was no significant association between BPA level and obesity.	9
Pornkunwilai et al., 2015 [19]	Cross-sectional	376	Thailand	3–18 y	BPA detection was significantly associated with obesity, but not with other demographic data or BPA exposure risks.	9
Valvi et al., 2013 [33]	Cohort	402	Spain	14 m–4 y	BPA exposure was weakly associated with obesity at 4 years.	7

^*^ Quality was assessed using NOS (Newcastle–Ottawa quality assessment scale) method.
